# Biomimetic Strategies for Sustainable Resilient Cities: Review across Scales and City Systems

**DOI:** 10.3390/biomimetics9090514

**Published:** 2024-08-27

**Authors:** Omar Borham, Ben Croxford, Duncan Wilson

**Affiliations:** 1 UCL Institute for Environmental Design and Engineering (IEDE), The Bartlett, UCL Faculty of the Built Environment, 14 Upper Woburn Place, London WC1H 0NN, UK; 2 UCL Centre for Advanced Spatial Analysis (CASA), University College London, London WC1E 6BT, UK

**Keywords:** biomimicry, sustainability, built environment, energy efficiency, resource efficiency

## Abstract

Biomimicry applications in different domains, from material science to technology, have proven to be promising in inspiring innovative solutions for present-day challenges. However, biomimetic applications in the built environment face several barriers including the absence of biological knowledge of architects and planners and the lack of an adequate common means to transfer biomimetic concepts into strategies applicable in the urban context. This review aims to create a multidimensional relational database of biomimetic strategies from successful precedent case studies in the built environment across different city systems and on different application scales. To achieve this, a thorough systematic search of the literature was implemented to map relevant biomimetic case studies, which are analyzed to extract biomimetic strategies that proved to be applicable and successful in an urban context. These strategies are then classified and documented in a relational database. This will provide a guide for architects and planners on how to transfer biomimetic strategies to strategies applicable in the urban context, thus bridging the gap of their lack of biological knowledge. The resulting matrix of strategies provides potential strategies across most of the different city systems and scales with few exceptions. This gap will be covered in a future work, currently in progress, to expand the database to include all city systems and scales.

## 1. Introduction

While cities represent just 3% of the Earth’s surface, they house over half of the world’s population, consume about 70% of global resources including energy resources, and account for three-quarters of the total greenhouse gas (GHG) emissions. These numbers are expected to worsen further since, according to a report by the United Nations, approximately two-thirds of the world’s population will move to urbanized areas by 2050 [1]. There is a need for rapid urbanization to meet the demands of the booming population worldwide, but at the same time, continuing down this path poses a huge threat to the environment, and as cities grow faster, they become more complex and harder to govern sustainably.

Cities have long been viewed as living organisms that require food to survive and that produce waste in a linear, non-cyclic process. Similarly, the urban metabolism in cities is linear, requires extensive energy, and produces waste. In the quest to rectify this, concepts of sustainability and eco-efficiency have emerged, but their application rather focuses on parts or elements in the built environment in isolation and attempts to improve them. This current thinking pattern turns a blind eye to the complex nature of cities with their many constituent systems and elements that affect one another and would, therefore, only provide approximate simplistic and reductionist solutions [2,3] instead of comprehensive solutions to environmental sustainability that will consider these complexities.

Therefore, a shift in mindset is needed to change the model by which cities are designed to a model of similar complexity and time-tested successes—nature. This is what biomimicry tries to achieve [4]. A breakdown of how natural ecosystems manage energy, water, and materials could provide insights into how cities can sustainably manage these resources, which sometimes require trade-offs within the system for the greater good.

This paper is a systematic review that focuses on applications of biomimetic strategies in the built environment—where nature’s organisms, processes, or ecosystems are mimicked in architectural and urban design—and the potential to present a novel approach for designing built environments to be truly sustainable or regenerative. The database collected and analyzed through this study and the ontology used to create relationships between the various examples will provide a valuable tool for city stakeholders, designers, and urban planners to create sustainable and resilient city systems.

## 2. Aim and Objectives

This paper aims to create a database that includes a selection of biomimetic strategies, based on patterns found in nature, from case studies in the built environment, which has a high potential of being useful to wider built environment applications. This review of the literature will help designers by increasing awareness of some specific applications and examples and categorizing them across scales, city systems, and biomimicry levels.

Accordingly, this study adopts the following objectives:

- A systematic review of previous literature identifying applications of biomimicry in the built environment is carried out.
- The most promising selected strategies and case studies of prior applications of biomimicry are assessed and ranked.
- A select database of biomimetic strategies is created and classified.

## 3. Background

**Figure 1:**
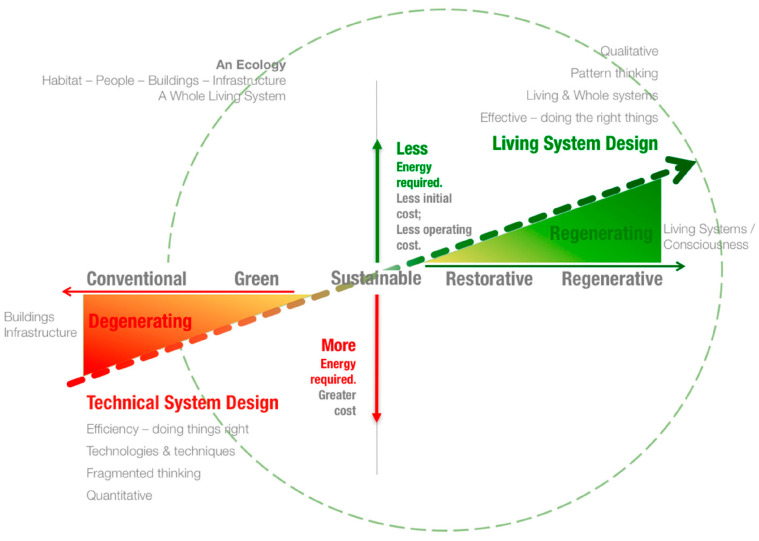
Trajectory of Ecological Design. Adapted with permission from reference [5].

The following sub-sections set out the definition of some high-level terms used in this systematic literature review. Figure 1 summarizes approaches to sustainability in the form of a line graph, where the differences in terms of impact can be seen from degenerating to regenerative, and also shows the energy required to carry out certain approaches, with less energy used being positive on the *y*-axis. The current conventional practices of sustainability lie on the degenerative side of the graph and are net energy users. This section discusses some more regenerative approaches to sustainability.

### 3.1. Regenerative Sustainability

Regenerative sustainability is about creating built environments that regenerate ecosystems and enable communities to thrive without ongoing intervention. It is a shift from a human-only oriented design that focuses on efficiency to a systems approach that acknowledges humans as an integral part of an ecosystem. Regenerative development aims to improve ecological health rather than degrade it and uses place-based, integrative, and participatory design methods to ensure significant community health and well-being benefits. A systems-based approach is crucial to regenerative development, allowing, for example, mutually beneficial interactions between the built environment, the living world, and human inhabitants over time.

The main challenges in implementing regenerative development are the current lack of an integrated approach and the scarcity of completed examples to provide quantifiable evidence of the benefits of regenerative built environments [6]. Regenerative design is described as “building capacity not things” where buildings are designed as systems that interact with each other, the living world, and their human inhabitants rather than as objects [5]. Leading thinkers on regenerative design argue that a shift from a built environment that ultimately degrades ecosystems to one that restores local environments and regenerates the capacity for ecosystems to thrive will require a fundamental rethinking of not just the architectural design [7] but also, as Hunt points out, rethinking the present competitive economic landscape of the built environment [8].

As explained in Table 1 below, despite the numerous benefits of a regenerative approach over conventional and eco-efficient approaches, it is faced with the challenge of its lack of compatibility with the current status quo and business-as-usual mindset.

### 3.2. Urban Resilience

The concept of resilience has a rich history across engineering, psychology, and disaster literature [9]. While various scholars have contributed to its development, ecologist C.S. Holling’s seminal paper in 1973 [10] is often regarded as the origin of modern resilience theory. Holling challenged the traditional ecological stability paradigm by recognizing ecosystems as dynamic and having multiple stable states. Resilience, according to Holling, describes an ecological system’s ability to persist and function even when altered without necessarily remaining unchanged [11,12,13].

Cities, like ecosystems, are not static and continuously evolve due to a variety of internal and external pressures (e.g., population growth, environmental changes, economic shifts, and technological advances. Urban resilience refers to the capability of an urban system, including its socio-ecological and socio-technical networks, to perform the following: 1. Maintain or rapidly return to desired functions after disturbances (e.g., natural disasters, social disruptions); 2. Adapt to ongoing changes (e.g., climate shifts); 3. Swiftly transform systems that currently limit adaptive capacity or hinder future resilience. This does not mean maintaining a status quo since cities (as in Holling’s resilience model) constantly undergo transformations [10].

Cities, being complex systems, require holistic approaches that consider interconnections among various domains. For example, a shock to the transportation system can affect economic productivity, social mobility, and even access to essential services. Therefore, planning for urban resilience must account for these interdependencies to ensure the city can withstand and adapt to disruptions while maintaining or quickly regaining desired functions even if the city’s structure or functions change. In this context, urban resilience serves as a boundary object, bridging expertise from multiple disciplines and stakeholders [14]. Therefore, implementing urban resilience involves diverse stakeholders with varying motivations, power dynamics, and trade-offs. Moreover, spatial and temporal scales play a crucial role in shaping resilience strategies [15]. This perspective means that urban planners should plan for adaptability, for the potential of transformation in ways that embrace change rather than merely resist it. This could mean rethinking urban infrastructure or governance models in the context of the interconnectedness of city systems [16].

### 3.3. City Systems and Flows

Globalization connects cities with distant places through material, energy, and capital exchanges [17,18]. This is mainly through trade and the movement of goods, fuel, and capital between distant cities. Nonetheless, each city in itself is a dynamic place where human and natural processes interact, forming urban ecosystems [19,20,21]. Cities can, therefore, be thought of as complex systems composed of interconnected subsystems [22].

City systems consist of four subsystems [10,21]:

1. *The physical built environment or the urban infrastructure and buildings:* Built environment, transportation, energy, water grids, and green spaces.
2. *Networked material and energy flows, also referred to as “metabolic flows”*: These include water, energy, food, materials, waste, and consumer goods.
3. *Governance Networks:* Actors and institutions shaping urban decisions such as consumers, NGOs, labor, industry, and the state.
4. *Socioeconomic Dynamics:* Social aspects influencing urban resilience like demographics, mobility, public health, capital, education, equity, and justice.

**Figure 2:**
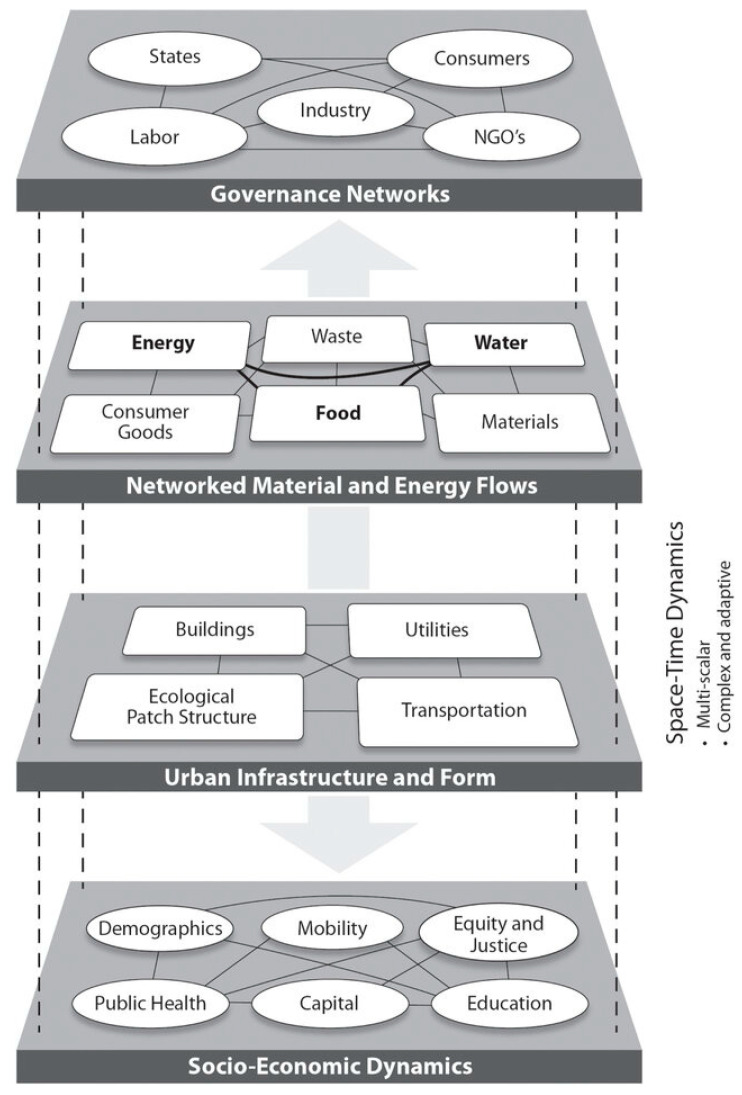
A simplified conceptual schematic of the multilevel networks within the city system. Reprinted with permission from reference [16].

Figure 2 provides a heuristic for understanding the intricate structures and dynamics of these urban systems [16]. The figure illustrates this concept of multilevel networks within the mentioned city systems. However, conventional design and governance often treat these subsystems as separate silos. This is due to the increasing specialization within the construction industry, which hinders holistic city-system interconnectivity. Transdisciplinary solutions require changes in city powers, cooperation, and mindset adaptation [10].

These subsystems interact at various scales (spatial and temporal), emphasizing interconnections [23,24,25]. For instance, by investing in wind turbines and biomass energy, a city can reduce its carbon footprint and improve air quality. This shift can also influence other systems, such as reducing the load on healthcare due to fewer respiratory issues from air pollution and encouraging economic growth in green technology sectors. Similarly, a well-functioning transportation system allows labor to commute easily, and it facilitates the movement of goods and services, thus increasing productivity and capital. Understanding such spatial and temporal interactions across networks is crucial for designing resilient cities. To comprehensively assess urban resilience, all these subsystems and their elements must be considered. This helps decision-makers think through the complexities involved in managing cities effectively.

### 3.4. What Is Biomimicry?

The term biomimicry was coined in 1997 by Janine Benyus. Biomimicry is from the Greek words “bios”, meaning “life”, and “mimesis”, meaning to “imitate”. Benyus, who is a biologist and a writer, defines it as “a new discipline that studies nature’s best ideas and then imitates these designs and processes to solve human problems” [4]. She explains that the field is grounded in the principles of ecological thinking and sustainability, where nature-inspired solutions are not only efficient but also have a low environmental impact.

Biomimicry has been adopted in a wide range of fields, from robotics and engineering to medicine and material science. Between each of these fields, the definition of biomimicry varies greatly. This is perhaps why Pederson Zari notes that there is no clear definition of biomimicry that architects could apply in designing their projects, and therefore, it is best to focus on analyzing the different approaches to biomimicry to come out with the best methods to apply biomimicry for maximum benefit [26]. Guber, on the other hand, defined biomimicry as “the study of overlapping fields of biology and architecture that show innovative potential for architectural problems” [27].

### 3.5. Levels of Biomimicry?

According to Benyus and Zari, there are three levels of biomimicry [28]. Firstly, if an organism’s form is mimicked, this is Organism level biomimicry. An example of this is the Lotusan paint, where the nanostructure of the lotus leaf’s surface was mimicked to create an engineered paint with similar surface properties that allow the paint to self-clean when subjected to the rain in a similar manner to the lotus leaves. The second level is behavior-level biomimicry, where an organism’s behavior or process is mimicked in design. This was the case with the Eastgate building in Zimbabwe, where the architect mimicked the way termites passively ventilated their mounds and created a similar ventilation mechanism that reduced the energy needed for artificial ventilation. Lastly, ecosystem-level biomimicry is when a design holistically mimics an entire ecosystem, including the complex links that relate to its components. HOK’s Lavasa masterplan proposal is an example of this level of biomimicry, where the hydrological cycle and the forest were analyzed and mimicked in the design of the buildings, landscape, and roads to create a city with minimum surface runoff and reducing the risk of flooding. The urban surfaces were designed to be permeable like a forest floor, while the buildings’ roof design was multilayered like a forest canopy to retain and re-evaporate rainwater back into the atmosphere. This is besides onsite rainwater collection and wastewater treatment and reuse for landscape irrigation. The latter, although seen as the ultimate goal, was, until lately, considered the most difficult form of biomimicry.

Kibert (2006) suggested that the complexity in understanding ecosystems makes it impossible for designers to engage in modeling ecosystems in their work since, according to Kibert, human designs are insufficiently complex. However, Zari argues otherwise. Zari defends that the ever-increasing knowledge about nature would enable us to mimic the complex relationships in ecosystems to increase the sustainability of the built environments [29].

There could be overlaps between the various levels of biomimicry, as are evident in the case studies handled in this review. For instance, several systems that relate to each other, such as in an ecosystem, are part of an ecosystem-level biomimicry. At the same time, the components of those systems may be modeled after organisms or their behavior in a similar way that a forest ecosystem is home to many interrelated organisms [26].

### 3.6. Nature’s Approach to Sustainable Design

**Figure 3:**
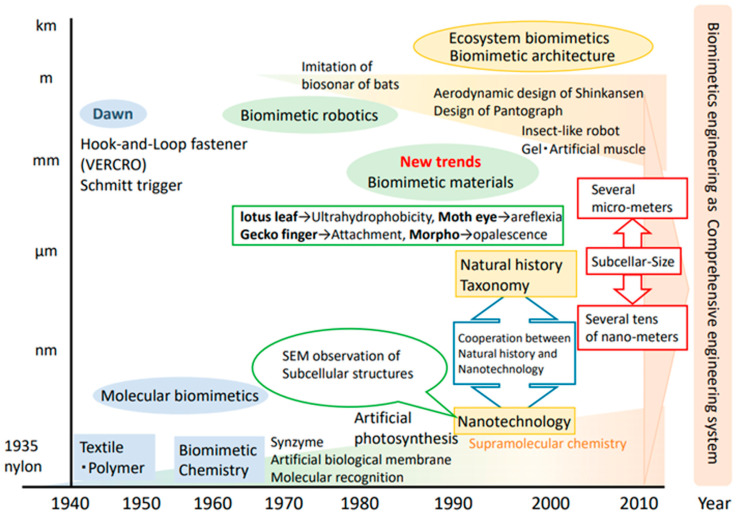
Historical trend of biomimicry across scales. Source [34].

Biomimicry involves applying nature’s design principles to human design. These principles were identified by Benyus [4] and were later refined by the Biomimicry Institute to include the use of only the energy needed, recycling of all materials, resilience to disturbances, optimization rather than maximization, reward for cooperation, use of information, use of safe chemistry and materials, use of abundant resources, being locally attuned and responsive, and using shape to determine functionality [30]. It is argued that the application of these principles in human designs would make these designs biomimetic; they would be sustainable and behave in a way similar to nature’s resilient designs. The concept of biomimicry can be applied to address challenges across various scales [31,32]. In built environment applications, this approach extends from the nanostructure of building materials to entire buildings and even urban areas that extend kilometers [33] (Figure 3).

### 3.7. Biomimicry in the Built Environment: Current State of Research

Although the approach of the application of biomimetic strategies is promising to reach regenerative built environments, the application of biomimicry in the built environment faces several challenges, according to various scholars. Firstly, there is a lack of a consistent and clear definition of biomimicry, which poses a challenge to understanding the implications of applying its abstract concepts [35]. Secondly, there is a gap in the availability of applicable methodologies to aid its incorporation into architectural and urban design [2,36]. Thirdly, there is a main concern that biological knowledge is not commonly accessible to architects and urban planners, which discourages them from trying to incorporate biomimicry in their designs [2]. This has led some scholars to suggest having a biologist on the design team in the early stages of the design process. Lastly, even if designers were aware of biological concepts, there are further knowledge barriers to transferring these concepts into designs and technologies that would be applicable within the built environment [33,37]. Although the awareness of the potential of biomimicry is increasing, it is still far from being common practice.

### 3.8. Contribution of the Study

This study is not concerned with providing a definition for biomimicry in the urban context. However, this paper is focused on addressing and bridging the rest of the gaps presented in the previous section by reviewing and selecting biomimetic strategies with high potential for their application to solve built environment problems. This study is focused on the mapping of successful precedent cases of the applications of biomimicry in the built environment on different scales. An analysis of the biomimetic strategies used and transferred to urban applications could overcome the barriers to the transferability of biomimetic concepts and bridge the gap of the lack of biological knowledge of architects and planners.

The classification and ontology presented in this study hope to offer a methodology for the comprehensive application of biomimicry in the built environment on various scales and across different city systems. For this purpose, different application scales were considered that varied from macro-scale, which this review refers to as urban scale, which includes applications of biomimicry on a scale larger than a building like a neighborhood, district, or even a city. This would be the scale that city planners or urban designers address in their designs. The second scale is the scale of a single building. This scale addresses the field of work of an architect. The final micro-scale is the scale of a building component or a building system within a building.

## 4. Materials and Methods

This review adopts an exploratory and analytic research methodology in an attempt to clarify the potential that lies in adopting biomimicry strategies to enable environmentally sustainable cities. The research follows a qualitative research design, which involves the collection and analysis of non-numerical data. The study starts with a systematic review approach where relevant scientific literature is reviewed, and appropriate case studies are analyzed. From this literature, biomimetic strategies are extracted to create a database of those that are most applicable in the built environment field. The review has been conducted in four phases.

**Figure 4:**
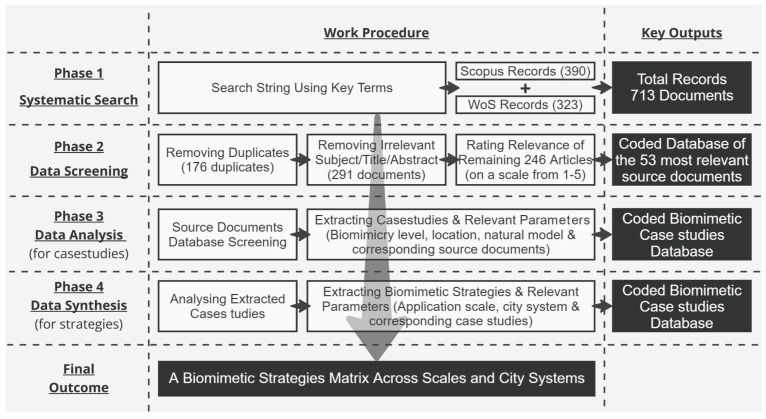
Process of systematic literature review. Source: designed by the author using Miro.

First, a systematic search is conducted to identify literature that studies applications of biomimicry in the built environment. Second, a screening process filters the results of the literature search according to the inclusion and exclusion criteria. Third, relevant case studies of prior applications of biomimicry from the chosen literature are collected and analyzed. Finally, a database of biomimetic, environmental, and sustainability strategies is created and classified according to application scale and relevant city system(s). The flowchart (Figure 4) summarizes the following four phases of this paper.

### 4.1. Phase 1: Data Collection

First, a comprehensive search of the literature was carried out using SCOPUS and Web of Science. The inclusion criterion was as follows:

- Literature search only using Web of Science and Scopus (Google Scholar was dismissed due to an anomaly in results, which produced an excessive number of irrelevant results);
- The combination of keywords as specified below in Table 2;
- Language: English;
- Published after 1997, when the term biomimicry was coined by Janine Benyus;
- Only peer-reviewed articles, conference papers, reviews, and books were selected, not magazine articles.

**Table 2:** Literature search parameters.

**Key terms**	- **Biomimicry** - Biomimetic strategies - Nature-inspired solutions - Sustainability - Resilience - Built environment
**Search string used**	TITLE-ABS-KEY ((“biomimetic” OR “biomimicry” OR “nature-inspired”) AND (“built environment” OR “architecture” OR “urban” OR “cities” OR “Buildings”) AND (“sustainable” OR “sustainability” OR “resilient”))
**Inclusion criteria**	- Scopus and Web of Science - Written in English - Published after 1997 - Peer reviewed - Strategies applicable in the built environment - Further papers were added following review of references of included papers.
**Exclusion criteria**	- irrelevant to the built environment - Duplicates

### 4.2. Phase 2: Data Screening

The results gathered during phase 1 were filtered using the following processes applied iteratively and in the following order:

- Titles and authors were arranged in a spreadsheet, allowing for sorting.
- Duplicates were identified and removed.
- Irrelevant documents according to title were removed.
- At this stage, abstracts were reviewed, and irrelevant documents were removed according to their abstracts.

The remaining documents were read and graded on relevance. The relevance scale was between 1 and 5. With 1, being the least relevant. Only those articles that were the most relevant (graded 5) were considered.

This selection process resulted in the 53 most relevant documents from a first search of 713 to be considered in detail as the source for extraction of biomimetic strategies with high potential to be useful to help solve built environment problems.

### 4.3. Phase 3: Data Analysis (for Case Studies)

In this phase, the researcher extracted strategies and summarized and synthesized the findings from the selected literature to create a database. In a Microsoft Excel spreadsheet, a table was created that included author(s), title, publication year, document focus, number of citations, document type, source title, and a coded description for each biomimetic case study mentioned in the source documents, note that some documents had several case studies. These coded case study descriptions were each assigned a unique identifier code prefixed by CS.

### 4.4. Phase 4: Data Synthesis (for Strategies)

A database of biomimetic strategies from the previous phase, which was used in the case studies extracted previously, was generated. These strategies, as well as other parameters extracted from the case studies that could be used to classify them, were added to a separate, linked database, which was a second Microsoft Excel spreadsheet. This database has a coded description for each strategy as well as other parameters, including built environment challenge, city system/flow involved, biomimicry level, and applied scale.

It was decided that although the 2017 list of Sustainable Development Goals and their indicators by the United Nations is comprehensive [38], limited indicators are relevant to city-level development. Therefore, city systems/categories included in the Green City Index were used instead to classify the different strategies as they are tailored to the built environment [39]. These categories/city systems are namely *Energy and Carbon*, *Water*, *Waste*, *Mobility and Transport*, *Infrastructure and Buildings*, *Food*, *Air Quality*, *Biodiversity and Green Infrastructure*, and *Governance and Data*.

Each strategy also had the case study code (CS###) from where the strategy was extracted. Each strategy was also given a unique identifier code with a prefix (S###). Some strategies were duplicated in several case studies.

The author continued reviewing and refining this strategy database until no further strategies were found. Through this process, a fully comprehensive list of high-potential strategies was found. This set of strategies was then further classified according to the application scale and the city systems.

## 5. Results

The data collection and screening phases of the methodology resulted in a list of 53 articles to be considered in the study, as summarized in Table 3 below. These are the source documents that will be used in the following tables to extract biomimetic case studies and strategies. The articles were ordered from the most cited articles to the least cited ones. The leftmost column contains the document ID number that will be used to identify each of the source document records. This identifying ID number will be used in Table 4 to link both tables together.

Each of these 53 articles was read and analyzed to extract the biomimetic case studies relevant to the built environment. Along with each case study, other parameters were also collected, including location, natural inspiration model, biomimicry level, and the corresponding source documents. These are summarized in Table 4 below. The leftmost column contains the case study ID (CS###) that will be used to identify each of the case studies. This identifying code will be used in Table 5 to link both tables (4 and 5) together. Moreover, to link each case study to the source documents in which they were mentioned, the source document ID(s) are provided in the rightmost column. This column acts as the link between both tables (3 and 4) to illustrate how they are related.

**Table 4:** List of biomimetic case studies extracted from the source documents in the previous table. **Abbreviations Legend** *Biomimicry Level: Organism Level (OL)*, *Behavior Level (BL)*, *Ecosystem Level (EL)*.

Case Study ID	Case Studies	Location	Natural Model	Biomimicry Level	Source Document ID(s)
CS001	Eastgate Building	Zimbabwe	Termite mound	BL	4, 8, 10, 17, 22, 23, 27, 32, 33, 37, 43, 46, 48, 50, 52
CS002	City Council House 2 (CH2)	Australia	Termite mound, trees bark	BL	4, 10, 22, 23, 32, 33, 37, 43, 46, 50
CS003	Lavasa	India	Indian Harvester Ant, Fig leaf, Natural water cycle, Ecosystem Performance Standards	BL, EL	9, 10, 19, 22, 26, 34, 40, 51, 52
CS004	Flectofins by ITKE	Stuttgart, Germany	Valvular pollination mechanism in the Strelitzia reginae flower (aka Bird-Of-Paradise flower)	OL	1, 3, 5, 17, 20, 27, 40
CS005	One Ocean Thematic Pavilion by SOMA Architecture	Yeosu, South Korea	Valvular pollination mechanism in the Strelitzia reginae flower (aka Bird-Of-Paradise flower)	OL	1, 3, 23, 27, 33
CS006	HygroSkin Pavilion	Orleans, France	spruce (pine?) cones passive response to humidity changes	OL	1, 3, 5, 17, 27
CS007	Lotusan Paint	Not Applicable	Lotus Leaves	OL	4, 8, 29, 39, 52
CS008	MMAA	Qatar	Cactus	OL, BL	22, 32, 43, 46, 48
CS009	Intitute de monde Arabe	France	Eye Iris	BL	4, 22, 27, 33
CS010	Water Cube National Swimming Center Beijing	China	Bubbles	OL	4, 10, 22, 27
CS011	Eiffel Tower	France	Thigh Bone	OL	10, 22, 23, 43
CS012	Pechino National Stadium (Birds Nest Stadium)	Beijing, China	Bird’s nest	OL	4, 10, 22, 27
CS013	Espalande theater	Singapore	Durian Fruit, sea urchin shells	OL	10, 22, 27, 33
CS014	Lloyd Crossing	USA	Local ecosystem patterns	EL	19, 40, 51, 52
CS015	Self-repairing concrete (Bio-concrete/Bionic self-healing concrete)	Not Applicable	Trees/fauna and human skin	BL	4, 37, 39
CS016	Calera Portland cement, Eco-Cement	Not Applicable	Salp fish, seashells, and the Saguaro cactus	BL	9, 37, 39
CS017	Urban Green Print Project	Seattle, USA	Water cycle, Forest	EL	9, 51, 52
CS018	Cooke’s koki’o photosensitive	Not Applicable	Photosynthesis, Cooke’s Koki`o (Kokia cookei)	BL	9, 37, 39
CS019	Living Machine/Eco-machine	Not Applicable	Natural water purification, Wetlands	EL	18, 37, 39
CS020	Lotus Temple	New Delhi, India	Lotus Flower	OL	22, 27, 33
CS021	Hydrological Center Namib University	Namibia	Stenocara Beetle	OL	35, 44, 52
CS022	IRLens Spot Heating System	Not Applicable	crayfish and lobster eyes	BL	37, 39, 50
CS023	Rafflesia Zero Energy House	Not Applicable	Rafflesia flower	BL	22, 33, 43
CS024	The Las Palmas Water Theater	Spain	Stenocara Namib Beetle	OL	4, 44
CS025	Heliotrope	Germany	Sunflower	OL	4, 17
CS026	Mobius	London, UK	ecosystem’s recycling of resources, Wetlands	EL	9, 20
CS027	Eco-Smart City of Langfang	Langfang, China	Natural water cycle, wetlands	EL	9, 26
CS028	Tensegrity (Kurilpa) Bridges	Australia	Spider web, human body’s adaptation to damage	OL, BL	9, 22
CS029	Biocement, Engineered cement composite	Not Applicable	flexible self-healing skin	BL	17, 48
CS030	i2 Modular Carpets	Not Applicable	Forest floor, organized chaos of nature’s ground coverings	OL	18,39
CS031	Explore Biomimetic office Building	Zurich, Switzerland	Spookefish eye, brittle starfish, Stone Plant, Bird’s skull, mimosa leaves, Beetle’s wings, mollusc’s iridescent shell, double-duty spinal column, mimosa pudica plant	OL, BL	23, 46
CS032	Sagrada Familia	Barcelona, Spain	Tree	OL	22, 27
CS033	Milwaukee Art Museum	Milwaukee, USA	Bird Wings, Animal bone	OL	4, 27
CS034	Eden Project	Cornwall, UK	Soap Bubbles Formation	BL	27, 33
CS035	Sahara Forest Project	Qatar, Tunisia, and Jordan	Namibian Desert Beetle, Ecosystem	BL, EL	24, 33
CS036	The carbon-neutral Utopian Village (coral reef project)	Haiti	Coral Reefs	EL	35, 43
CS037	BioWave	Not Applicable	Bull Kelp, Cochayuyo seaweed withstand strong wave forces by being flexible and stretchy	OL	37, 39
CS038	Biolytix System	Not Applicable	Earth Ecosystem	EL	37, 39
CS039	COMOLEVI Forest Canopy	Not Applicable	Shadow Trees	OL	37,39
CS040	Sage GlassQuantum Glass	Not Applicable	Bobtail squid, hummingbird	OL	37, 39
CS041	Aquaporin Membrane	Not Applicable	lipid bilayer of living cells, cell membrane	BL	37,39
CS042	Chaac-ha	Not Applicable	Spiders and Bromeliads	OL	37, 39
CS043	Purebond (Bioplywood)	Not Applicable	Blue mussel mollusk adhesion	OL	37, 39
CS044	Gherkin Tower, SwissRe Headquarters	London, UK	Venus flower basket sponge	OL	22, 43
CS045	Encycle BMS Swarm Logic	Not Applicable	Honeybees	BL	23, 50
CS046	Waterloo International Terminal	Waterloo, UK	pangolin	OL	22, 53
CS047	brewery near Tsumeb	Namibia	Ecosystem	EL	2
CS048	Sunflower fiber optic lighting system	Japan	Sunflower	OL	4
CS049	Urban Cactus	Netherlands	phyllotaxy, which refers to the way in which the leaves of different plants grow on the stem and which varies between alternate phyllotaxy	OL	4
CS050	Haikou Tower	China	fins	OL	4
CS051	Duisburg Business Support Cente	Germany	biological circulatory system	OL	4
CS052	The Sky house by kiyonori Kikutake	Japan	Growth and Metabolism	BL	4
CS053	Tokyo Dome Stadium	Japan	Bubbles	OL	4
CS054	School of Youth Education designed by Thomas Herzog	Germany	Polar Bear Skin	OL	4
CS055	Self-cleaning traffic light glass	Germany	Lotus Leaves	OL	4
CS056	Willis Tower	Chicago, USA	Bamboo	OL	4
CS057	BMW Office Building	Munich, Germany	Ears of wheat	OL	4
CS058	Rome Gatt Wool Factory	Italy	Lotus leaf vein	OL	4
CS059	Worker’s Stadium	Beijing, China	Cobweb	OL	4
CS060	Fuji Pavilion World Expo, 1970	Osaka, Japan	Soap bubble	OL	4
CS061	National Industries & Techniques Center	France	Eggshell	OL	4
CS062	The Montreal Biosphere	Montreal, Canada	Honeycomb	OL	4
CS063	Palazzeto Dellospori	Rome, Italy	Amazon Water Lilly	OL	4
CS064	Albufeira River Restoration	Portugal	Nature Based Solutions, Soil, Evapotranspiration	EL	6
CS065	Van Gogh Roosegaarde cycle route	Eindhoven, Netherlands	Bioluminescence	BL	6
CS066	Tokyo railway mapping experiment	Tokyo, Japan	Physarum polycephalum Slime Mould	BL	9
CS067	Wellington	New Zealand	Ecosystem services (provision of water and energy)	EL	17
CS068	Green surge project	Europe	Nature	EL	17
CS069	Kalundborg Industrial Complex	Kalundborg, Denmark	ecosystem’s recycling of resources	EL	18
CS070	Organic Waste Biodigester	Not Applicable	Natural Decomposition Process	BL, EL	18
CS071	Bullet train	Japan	Kingfisher Bird’s beak	OL	20
CS072	Silk Pavilion	Massachusetts, USA	Silkworm	OL	20
CS073	Biohaven’s Floating Islands	Not Applicable	Wetland ecosystems	EL	20
CS074	Sinosteel International Plaza	Tianjin, China	Beehive	OL	22
CS075	Habitat 2020	Not Applicable	stomata of leaves	BL	22
CS076	Tree scraper, tower of tomorrow	Not Applicable	Tree growth	BL	22
CS077	Taichung Opera house	Taichung, Taiwan	Schwarz P type	OL	22
CS078	Earth ships	Not Applicable	Ship?	EL	22
CS079	Treepods	Boston, USA	Dragon tree	BL	22
CS080	All seasons tent tower	Armenia	Mt. Ararat	OL	22
CS081	Lily pad floating city	Not Applicable	Lily pad	EL	22
CS082	Loblolly House	Maryland, USA	tree house	BL	22
CS083	Shi ling bridge	China	shell lace structure	OL	22
CS084	Guggenheim Museum	New York, USA	Ship	OL	22
CS085	Parkroyal	Singapore	Vertical Garden	BL	22
CS086	SUTD library pavilion	Singapore	timber shell	BL	22
CS087	Sydney opera house	Sydney, Australia	shell structure	OL	22
CS088	Redwood Tree house	New Zealand	seed pod	OL	22
CS089	TWA terminal	New York, USA	bird flight	OL	22
CS090	Institute for Computer-Based Design	Stuttgart, Germany		BL	23
CS091	Himalayan rhubarb towers	China	Metabolism heat	BL	23
CS092	Cabo Llanos Towers	Santa Cruz de Tenerife, Spain		BL	23
CS093	Simon Center for Geometry and Physics at the State University	New York, USA	Tree Canopy	OL	23
CS094	Hobermann’s Dynamic Windows	Not Applicable	Tree Canopy	OL	23
CS095	phyllotactic towers	Iran	Plants with phyllo-tactic geometry	OL	23
CS096	Pantheon	Rome, Italy	Seashell	OL	23
CS097	Vertical Wind turbines	Not Applicable	Schools of fish	BL	23
CS098	humpback fin wind turbine	Not Applicable	humpback whale fin	OL	23
CS099	Green Power Island	Not Applicable	Energy storage	BL	23
CS100	Max Fordham’s House	London, UK	Metabolism heat	BL	24
CS101	IKEA’s Space 10 lab miniature wooden village	Copenhagen, Denmark	Mycellium	BL	24
CS102	Here East	Lonon, UK	Nature recycles everything	EL	24
CS103	Waterloo City Farm	Waterloo, UK	Nature recycles everything	EL	24
CS104	Rieselfeld & Vauban	Freiburg, Germany	Ecosystem	EL	25
CS105	Hammarby Sjostad District	Sweden	ecosystem’s recycling of resources	EL	25
CS106	Crystal Palace	London, UK	Victoria amazonica	OL	27
CS107	Teatro del Agua	Canary Islands	Stenocara Beetle, Hydrological cycle	BL	28
CS108	Self-cleaning Solar Panels	Not Applicable	Lotus Leaves	OL	29
CS109	Homeostatic Façade	New York, USA	Muscles	BL	33
CS110	Cairo Gate Residence	Cairo, Egypt	Termite Mound	BL	33
CS111	Durban resilient development plan	South Africa	Kwazulu Natal-Cape coastal forests, Southern Africa mangroves	EL	34
CS112	Interface Inc.: factory as a forest	Lagrange, USA	Oak–hickory–pine forest	EL	34
CS113	Adaptive fitting glass	Not Applicable	Namaqua chameleon	BL	35
CS114	Dockside Green development	B.C, Canada	Hydrological cycle	EL	36
CS115	Vancouver Olympic Village at Southeast False Creek	Vancouver, Canada	Hydrological cycle	EL	36
CS116	Radiant Cooling Technology	Not Applicable	Ground water channels	EL	37
CS117	Turtle glass	Not Applicable	Chelonia mydas	OL	37
CS118	sharklet	Not Applicable	Shark skin	OL	39
CS119	Lotus clay roofing tiles	Not Applicable	Lotus Leaves	OL	39
CS120	Ornilux insulated glass,	Not Applicable	Orb weaver spiders	OL, BL	39
CS121	BioUrban 2.0	Panama City, Panama	Trees	BL	40
CS122	Photocatalytic cement	Milan, Italy	nature uses nonharmful chemicals	EL	40
CS123	IONITY	Europe	Nature uses clean energy	EL	40
CS124	Sierpinski roof	Not Applicable	Sierpinski forest	OL	40
CS125	La Paz and El Alto	Bolivia	ant colony algorithm	BL	40
CS126	Plus-energy Rooftop Unit	Not Applicable	Liana	BL	41
CS127	CSET building	Ningbo, China	natural flows	EL	42
CS128	Pearl River Tower	China	Sea sponge	OL	43
CS129	Warka Towers	Ethiopia	Spider Web	OL	44
CS130	Rainbellows	Seattle, USA	Ice Flower	OL	44
CS131	The Media TIC building	Barcelona, Spain	Stomata	BL	46
CS132	Doha Tower	Doha, Qatar	Cactus Pores	BL	46
CS133	Tricon Corporate Center	Lahore, Pakistan	Oxalis Oreganada leaf	BL	46
CS134	Al Bahar Tower	Abu Dhabi, UAE	White Butterfly	BL	46
CS135	Model Community at Salton Sea	California, USA	Ecosystem, Algae	EL	47
CS136	MemBrain blocks	Not Applicable	stomata transpiration	BL	48
CS137	Zira Island	Azerbaijan	Forest Ecosystem	EL	48
CS138	Davis Alpine House in Kew Gardens	London, UK	termite mound	BL	52
CS139	Hemisferic	Valencia, Spain	Eyelid	OL	27

An analysis of each case study produced one or more entries in the database, as shown in Table 5; these are the biomimetic strategies that were used in each preceding case. Strategies were categorized according to a number of parameters, including city systems (*Energy and Carbon*, *Water*, *Waste*, *Mobility and Transport*, *Infrastructure and Buildings*, *Food*, *Air Quality*, *Biodiversity and Green Infrastructure*, and *Governance and Data*) and application scale (*Urban Scale*, *Building Scale*, and *Building Component Scale*). These parameters were added in the two rightmost columns. These parameters will play a role in the classification process of the strategies to form a framework for applying them in the built environment across different scales and city systems. Moreover, to link each biomimetic strategy to the corresponding precedent case study or case studies, the case study ID(s) are provided in the middle column. This column acts as the link between both tables to illustrate how they are related.

Each strategy was given an identification code (S###) to facilitate referencing it in the rest of the study. The strategies above represent a set of biomimetic strategies that were applied in the built environment in precedent case studies and can, therefore, represent a guide to architects and planners on how to apply biomimicry in the built environment. However, these applications are on different scales in the built environment. A fraction of these strategies can be applied on a scale as large as a district, neighborhood, or even a city, while others can be applied to a building or a building component. Moreover, the strategies target different aspects of city systems such as energy efficiency, water conservation, material efficiency, data flow, and biodiversity.

## 6. Discussion

The outcome of the methodology applied is a relational database consisting of three tables (Table 3, Table 4 and Table 5) which are linked together. Table 5 provides the biomimetic strategies that are applicable in the built environment for the design of regenerative, resilient cities, and these strategies are applicable on different scales and through different city systems. Table 4 provides a mapping of best-practice biomimetic case studies in the built environment. The precedent application of any of the strategies from Table 5 can be found in one or more precedent case studies, which can provide a guide to architects and planners on how to successfully apply a certain strategy to the built environment. Therefore, Table 5 and Table 4 were linked together via the case study identification code (CS###) to cross-reference the strategies with their corresponding precedent case studies. Where more information is needed by the architect or planner about a particular case study or a strategy, the source document(s) in Table 3 can be consulted. Accordingly, Table 3 and Table 4 were linked together via the source document ID number to cross-reference the case studies with their corresponding source documents in which they were mentioned and analyzed. How best to organize and use this relational database is the subject of current research by the authors, as will be discussed in the Future Work section.

The diverse parameters included in the database allowed for a multidimensional set of strategies that could be filtered and reordered to provide insights on how to tackle a particular dimension. For instance, all strategies related to energy could be filtered to provide a set of strategies that focus on minimizing energy consumption. Another dimension could be filtering all strategies relating to the applied scale to give guidelines on how to design sustainable buildings, for example. This ontology allows this database to be used in different ways that correspond to the different goals of the architect or planner using the database. This filtering across the application scales and city systems can act as a valuable tool for architects and planners and facilitate the application of biomimetic strategies in the built environment.

An indication of how this will work can be summarized in the following matrix (Table 6), where all biomimetic strategies applicable on a particular scale can be found in the same column, while all strategies addressing a particular urban system can be found in the same row. This classification can make it more accessible to architects and planners to decide on which scale they are tackling and what challenges they are trying to address in their designs, who can, therefore, refer to the relevant biomimetic strategies after getting the strategy codes (S###) from the matrix of Table 6 and cross-referencing them to Table 5 to find the corresponding strategy. For further information on how to apply these strategies, the next step would be to cross-reference the strategies with the corresponding precedent case studies (in Table 4), as explained earlier using the case study ID(s) (CS###). For further details on a case study, a further step would be to cross-reference the case study to the relevant source document in Table 3 using the corresponding source document ID(s).

For example, to consider water use at the building scale, one might review one of the five strategies relevant strategies in Table 6 below. If the architect referring to the matrix decides to consider applying strategy S149; fog-catching (highlighted in bold in the matric below), the architect can refer to the relational database for ideas on how to apply this in the building design. Strategy S149 is linked to case studies CS020, CS021, CS024, CS035, CS042, CS107, and CS129 and the source documents 4, 22, 24, 27, 28, 33, 35, 37, 39, 44, and 52. These documents along with the case studies will provide a detailed guide on how to apply fog-catching technologies on the building scale and present previous successes.

It is observed in Table 6 that some cells were empty, presenting no strategies for a particular city system on a particular application scale. This can be due to two reasons. Firstly, certain challenges may only be tackled on a particular scale. For instance, all strategies collected for *Mobility and Transport* were on the *urban scale* and none on the other two scales. This can be understood since transport is usually between distant places normally on a scale larger than one building. Secondly, it could be that the collected source documents lacked case studies or strategies for that particular city system and scale, as will be explained in the Limitations section. An example of this is the lack of strategies for *Food* on the *building scale* or the *building component scale*. One might argue that a productive green roof can be a potential strategy to fill this gap, but such a strategy was missing from the collected source documents. The Future Work section will outline how this will be overcome in further research after this study.

## 7. Conclusions

Architects and planners require great awareness to achieve efficiency and sustainability in buildings, especially in this era when meeting sustainability targets is more critical than in the past. Architects and planners need to rethink the way they build in order to achieve a truly sustainable future. Novel ideas need to be explored and tested; a new design model is needed. The natural world provides an extensive design database that can inspire solutions as sustainable, resilient, and self-perpetuating as those seen in nature. The case studies and strategies presented here are successful precedents biomimetic applications in the built environment. Accumulating and presenting them in this relational database can help to develop wider awareness and understanding of the potential of biomimicry, which can help develop cities that are regenerative and resilient.

The design and management of future cities could also incorporate biomimicry but may have significant barriers due to the wider transdisciplinary nature of the field. While it is true that there is a gap in the biological knowledge of architects and planners, this gap can be bridged by providing a database of biomimetic strategies that have already been successfully applied in the built environment, along with the precedent case studies and source documents to support the understanding of how biomimicry applications can be transferred to the built environment.

Biomimicry, the science of imitating natural models, has good potential when integrated into the design of the built environment. While Benyus suggests that “a full emulation of nature engages at least three levels of mimicry: form, process, and ecosystem” [4], the authors propose a comprehensive vision of applying biomimicry in the built environment on different scales and across all city systems to achieve a regenerative, resilient city.

Ecosystem-level biomimicry gives a more holistic approach to the design of built environments. If applied on an urban scale, it would allow designing better cities that behave like natural ecosystems, and within those ecosystems, architects could also design buildings and building systems that thrive in themselves to achieve higher levels of efficiency in terms of energy, water, and resource use. Moreover, how natural ecosystems respond to place and the local environment is very important in setting design goals in terms of energy, air, water, and carbon budgets for a given design to ensure that cities can behave as a natural ecosystem would behave.

## 8. Limitations and Future Work

The authors acknowledge that this database is of limited scale, but it is expandable as more strategies or case studies are found. It is also noted that the resultant number and variety of strategies and case studies depended on the criteria put forward to limit the number of source documents that were reviewed. Widening the search scope in the future to include more articles will result in a richer database of case studies and strategies that better cover areas lacking in the biomimetic strategies matrix presented in Table 6.

Since this database is a work in progress that is set to be expanded and developed further in the future, it will be materialized into a digital application to facilitate access to the database. This would also allow for the accumulation of more biomimetic best practice cases in the built environment from architects and planners using this platform.

## Figures and Tables

**Table 1:** Benefits of eco-efficient and regenerative design. Source: [7].

	Conventional	Eco-Efficiency	Regeneration
Works within the existing mindset	✓	✓	
Minimizes environmental impact		✓	✓
Enhances people’s physical well-being		✓	✓
Boosts psychological health		✓	✓
Reduces overall lifecycle costs		✓	✓
Enhances economic value in projects		✓	✓
Fosters innovation in projects		✓	✓
Yields positive environmental outcomes			✓
Transforms development into a potential income source			✓
Manages global issues strategically via place-based approaches			✓
Improves integrated knowledge of place			✓
Promotes mutually beneficial relationships between people and place			✓
Enhances resilience, flexibility, and adaptability in built environments			✓
Strengthens equitable communities			✓

√: benefits.

**Table 3:** List of scientific articles relevant to biomimetic applications in the built environment. Full list of the source documents is available upon request from the author. For readability reasons, the table has been formatted with shortened references.

SourceDoc ID	Author, Year	Title	Source Document Focus	Citation Ref-No
1	(López et al., 2017)	How plants inspire facades. From plants to architecture: Biomimetic principles for the development of adaptive architectural envelopes	Adaptive building envelopes	[40]
2	(Mathews, 2011)	Towards a Deeper Philosophy of Biomimicry	Philosophical principles	[41]
3	(Al-Obaidi et al., 2017)	Biomimetic building skins: An adaptive approach	Adaptive building envelopes	[42]
4	(Yuan et al., 2017)	Bionic building energy efficiency and bionic green architecture: A review	Energy efficiency, structure, and materials	[43]
5	(Anzaniyan et al., 2022)	Design, fabrication and computational simulation of a bio-kinetic façade inspired by the mechanism of the Lupinus Succulents plant for daylight and energy efficiency	Biomimetic kinetic envelope design	[44]
6	(Blau et al., 2018)	Urban River Recovery Inspired by Nature-Based Solutions and Biophilic Design in Albufeira, Portugal	Nature-based solutions	[45]
7	(Hayes et al., 2019)	Leveraging socio-ecological resilience theory to build climate resilience in transport infrastructure	Transport infrastructure	[46]
8	(Ahamed et al., 2022)	From biology to biomimicry: Using nature to build better structures-A review	Envelopes, structure, and materials	[47]
9	(Buck, 2017)	The art of imitating life: The potential contribution of biomimicry in shaping the future of our cities	City systems	[48]
10	(Radwan & Osama, 2016)	Biomimicry, An Approach For Energy Efficient Building Skin Design	Buildings envelopes	[49]
11	(Hayes et al., 2020)	Learning from nature—Biomimicry innovation to support infrastructure sustainability and resilience	Structure and infrastructure	[50]
12	(Zari & Hecht, 2020)	Biomimicry for Regenerative Built Environments: Mapping Design Strategies for Producing Ecosystem Services	Ecosystem services	[51]
13	(Gruber & Imhof, 2017)	Patterns of Growth-Biomimetics and Architectural Design	Growth patterns	[52]
14	(Badarnah, 2015)	A Biophysical Framework of Heat Regulation Strategies for the Design of Biomimetic Building Envelopes	Envelopes (heat regulation)	[53]
15	(Chou et al., 2016)	Big data analytics and cloud computing for sustainable building energy efficiency	Energy efficiency management	[54]
16	(Pedersen Zari & Koner, n.d.)	An ecosystem based biomimetic theory for a regenerative built environment	Ecosystem principles	[55]
17	(Uchiyama et al., 2020)	Application of biomimetics to architectural and urban design: A review across scales	Biomimicry across scales	[33]
18	(Carlos Montana-Hoyos & Carlos Fiorentino, 2016)	Bio-utilization, bio-inspiration, and bio-affiliation in design for sustainability: Biotechnology, biomimicry, and biophilic design	Education	[56]
19	(Blanco et al., 2021)	Urban Ecosystem-Level Biomimicry and Regenerative Design: Linking Ecosystem Functioning and Urban Built Environments	Ecosystem biomimicry	[57]
20	(Ilieva et al., 2022)	Biomimicry as a Sustainable Design Methodology-Introducing the ‘Biomimicry for Sustainability’ Framework	Classification framework	[58]
21	(Badarnah, 2016)	Light management lessons from nature for building applications	Light management	[59]
22	(Dash, 2018)	Application of biomimicry in building design	Case studies classification	[60]
23	(Jamei & Vrcelj, 2021)	Biomimicry and the Built Environment, Learning from Nature’s Solutions	Envelopes, structure, materials, and energy retrofits	[61]
24	(Timea Kadar & Manuella Kadar, 2020)	Sustainability Is Not Enough: Towards AI Supported Regenerative Design	AI for regenerative design	[62]
25	(Spiegelhalter & Arch, 2010)	Biomimicry and circular metabolism for the cities of the future	Ecosystem biomimicry	[63]
26	(Lazarus & Crawford, n.d.)	Returning genius to the place	Ecosystem biomimicry	[64]
27	(Sommese et al., 2022)	A critical review of biomimetic building envelopes: towards a bio-adaptive model from nature to architecture	Adaptive building envelopes	[65]
28	(Pedersen Zari, 2009)	An architectural love of the living: Bio-inspired design in the pursuit of ecological regeneration and psychological well-being	Ecosystem biomimicry	[66]
29	(Dicks et al., 2021)	Applying Biomimicry to Cities: The Forest as Model for Urban Planning and Design	Forest ecosystem biomimicry	[67]
30	(Faragalla & Asadi, 2022)	Biomimetic Design for Adaptive Building Facades: A Paradigm Shift towards Environmentally Conscious Architecture	Adaptive building envelopes	[68]
31	(Imani & Vale, 2022)	Developing a Method to Connect Thermal Physiology in Animals and Plants to the Design of Energy Efficient Buildings	Thermal energy efficiency	[69]
32	(Faragllah, 2021)	Biomimetic approaches for adaptive building envelopes: Applications and design considerations	Adaptive building envelopes	[70]
33	(Verbrugghe et al., 2023)	Biomimicry in Architecture: A Review of Definitions, Case Studies, and Design Methods	Biomimetic design methods	[37]
34	(Benyus et al., 2022)	Ecological performance standards for regenerative urban design	Ecological performance standards (EPS)	[71]
35	(Elshapasy et al., 2022)	Bio-Tech Retrofitting To Create A Smart-Green University	Biomimicry and smart buildings	[72]
36	(Hao et al., n.d.-b)	Closed-Loop Water and Energy Systems: Implementing Nature’s Design in Cities of the Future	Closed-loop urban water systems	[73]
37	(Movva & Velpula, 2020)	An analytical approach to sustainable building adaption using biomimicry	Building scale biomimetic design	[74]
38	(Hao et al., 2010a)	Network Infrastructure—Cities of the Future	Urban water management	[73]
39	(Oguntona & Aigbavboa, 2019)	Assessing the awareness level of biomimetic materials and technologies in the construction industry	Biomimetic construction materials and technologies	[75]
40	(Quintero et al., 2021)	Sustainability Assessment of the Anthropogenic System in Panama City: Application of Biomimetic Strategies towards Regenerative Cities	Biomimetic regenerative cities and EPS	[76]
41	(Speck et al., 2022)	Biological Concepts as a Source of Inspiration for Efficiency, Consistency, and Sufficiency	Biological concepts of lianas	[77]
42	(Widera, 2016)	Biomimetic And Bioclimatic Approach To Contemporary Architectural Design On The Example Of CSET Building	Biomimicry for net zero buildings	[78]
43	(AlAli et al., 2023)	Applications of Biomimicry in Architecture, Construction and Civil Engineering	Biomimicry in building design	[79]
44	(Aslan et al., 2022)	A Biomimetic Approach to Water Harvesting Strategies: An Architectural Point of View	Water harvesting on the building level	[80]
45	(Ortega Del Rosario et al., 2023)	Environmentally Responsive Materials for Building Envelopes: A Review on Manufacturing and Biomimicry-Based Approaches	Responsive building envelopes	[81]
46	(Elsakksa et al., 2022)	Biomimetic Approach for Thermal Performance Optimization in Sustainable Architecture. Case study: Office Buildings in Hot Climate Countries	Envelope Thermal Performance	[82]
47	(Mazzoleni et al., 2008b)	Eco-systematic restoration: a model community at Salton Sea	Biomimetic urban Restoration	[83]
48	(Sharma & Singh, 2021)	Protecting humanity by providing sustainable solution for mimicking the nature in construction field	Biomimicry levels in built environment	[84]
49	(Van Den Dobbelsteen et al., 2010)	Cities As Organisms: Using Biomimetic Principles To Become Energetically Self-Supporting And Climate Proof	Biomimetic city planning principles	[85]
50	(Pedersen Zari M, 2018)	Can built environment biomimicry address climate change?	Biomimetic strategies	[7]
51	(Pedersen Zari M, 2018)	Emulating ecosystem services in architectural and urban design Ecosystem services analysis	Ecosystem services	[7]
52	(Pedersen Zari M, 2018)	Incorporating biomimicry into regenerative design	Biomimetic strategy regenerative design	[7]
53	(Pedersen Zari M, 2018)	Translating ecosystem processes into built environment design	Ecosystem services	[7]

**Table 5:** List of biomimetic strategies extracted from the precedent biomimetic case studies. **Abbreviations Legend** *Application Scale: Urban Scale (U)*, *Whole Building (B)*, *Building Component (C)*. *City Systems*: *Energy and Carbon (EC)*, *Water (WR)*, *Waste (WS)*, *Mobility and Transport (MT)*, *Infrastructure and Buildings (IB)*, *Food (FD)*, *Air Quality (AQ)*, *Biodiversity and Green Infrastructure (BG)*, *Governance and Data (GD)*.

Strategy ID	Biomimetic Strategy	Corresponding Case Study ID	Application Scale	City Systems
S001	Sequester atmospheric carbon into building materials, Neutral and strength-enhancing carbon sequestering cement	CS003, CS016, CS111, CS112	C	EC, IB
S002	Low Carbon Economy (LCE)	CS105	U	EC
S003	(Efficient) wind turbines	CS097, CS098, CS064, CS104, CS110, CS127, CS128, CS135	U, C	EC
S004	Hydro turbines	CS036, CS037	U	EC
S005	Geothermal energy	CS104	U	EC
S006	CHP—Combined Heating and Power Plants	CS104	U	EC
S007	Solar Photovoltaic Panels (on building’s roof and façade)	CS023, CS036, CS064, CS076, CS080, CS104, CS110, CS126, CS127, CS135	B	EC, IB
S008	Dye-Sensitive Solar cells	CS018	C	EC
S009	Solar Benches	CS064	U	EC
S010	Solar light posts	CS064	U	EC
S011	Biofuel producing algae farms	CS135	U	EC, BG
S012	Biomass	CS104	U	EC
S013	Blue battery, energy storage for different RE outputs	CS099	U	EC
S014	Batteries to store renewable energy	CS078, CS135	B	EC
S015	Bioluminescence Materials	CS065	U	EC
S016	P2P energy sharing via blockchain technology	CS101	B, C	EC, GD
S017	Reduce Peak Demand	CS045	C	EC, GD
S018	Zero (fossil) energy	CS023, CS104, CS135	B	EC
S019	low energy passive house	CS104	U	EC, IB
S020	Passive design strategies	CS135, CS104	U	EC, IB
S021	Active Solar design strategies	CS104	U	EC, IB
S022	Wall/slab thermal mass	CS135	U	EC, IB
S023	Energy excess fed into grid	CS135	U	EC
S024	Double glazing	CS135	U	EC, IB
S025	Openings sizing to control solar radiation	CS135	U	EC, IB
S026	District Heating/Cooling	CS104	U	EC
S027	Spot heating system	CS022	C	EC
S028	Underground radiant heating/cooling	CS002, CS051, CS116, CS135	C	EC
S029	Geothermal heat pump	CS110, CS127, CS135	B, U	EC
S030	Cooling by avoiding direct sunlight	CS092	B	EC
S031	Radiative heat gain	CS092	B	EC
S032	Heat by Occupants’ Metabolism	CS091, CS100	B	EC
S033	Improved Trombe wall	CS054	C	EC
S034	Solar water heating/Solar Collector	CS025, CS127, CS135	B	EC
S035	Sewage heat recovery	CS115	U	EC
S036	Heat sinks	CS135	U	EC
S037	Solar ponds	CS135	U	EC
S038	Water cooled façade	CS135	U	EC, IB
S039	Passive Cooling (Stack effect Ventilation)	CS001, CS002, CS138	B	EC
S040	Natural Cross Ventilation	CS050, CS135	B	EC, AQ
S041	Demand-driven ventilation system	CS085	B	EC, AQ
S042	Wind Catchers	CS023, CS110	B	EC, IB
S043	Minimal Structural members for maximum daylight	CS034	B	IB, EC
S044	Fiber optic lighting system	CS048	C	EC, IB
S045	Phyllotaxy/Fibonacci order to avoid self-shading	CS049, CS095	B	IB, EC
S046	Narrow Floor Plan Depth	CS031, CS135	B	IB, EC
S047	Reflect/Focus light into Dim Areas	CS031	B, C	EC, BI
S048	Inflatable membrane structures	CS010, CS053, CS060	B	IB, EC
S049	Responsive Adaptive skin color change to retain or absorb heat	CS113	B	EC, IB, GD
S050	Solar Envelope Masterplanning	CS104	U	EC, IB
S051	Elastically Deformable Louvers	CS004, CS005	C	EC, IB
S052	Solar Self-Shading	CS008, CS013, CS039, CS074, CS080, CS093, CS124	B	EC, IB
S053	Responsive Adaptive Shading System	CS009, CS031, CS134	C	EC, GD
S054	Kinetic screen	CS132	B	EC, GD
S055	Foldable Shading Devices	CS031	B, C	EC
S056	Adjustable Shading Device	CS133, CS033, CS139	B	EC
S057	inflatable shading device	CS131	C	EC
S058	Dynamic Windows	CS094	C	EC
S059	Electrochromic smart windows for energy savings	CS040	C	EC, GD
S060	Dyed glass to decrease light projection	CS117	C	EC
S061	Self-thermoregulation hybrid systems	CS127	B	EC, GD
S062	Responsive Adaptive envelopes	CS090, CS109	B, C	IB, EC
S063	humidity-sensitive envelope	CS006	C	IB
S064	Envelope controls daylight and air quality	CS075	B	EC, IB
S065	Walkable city/compact city design	CS003, CS104	U	MT, EC, AQ
S066	Building on columns for less footprint	CS023	B	IB
S067	Allow for Growth (degrowth)	CS052	B	IB
S068	Design for disassembly	CS062, CS082, CS103	B	IB
S069	Standardized modular prefabricated parts	CS030, CS036, CS062, CS082	C	IB
S070	Refurbish rather than dismantle	CS102	B	IB
S071	Design for Longevity	CS102, CS103	B	IB
S072	Design for adaptability	CS103	U	IB
S073	Adaptive Building Zoning	CS025	B, C	IB, EC
S074	Reduce surface area to volume ratio	CS076	B	IB, EC
S075	Parasitic Architecture (addition of net zero units on top of existing buildings, surplus PV power provided to the building in exchange for use of staircase, etc.)	CS126	B	IB
S076	Building orientation	CS135	U	IB, EC
S077	Decentralization	CS104	U	GD
S078	Decentralized services and markets	CS104	U	IB
S079	Hexagonal structural elements	CS074, CS053, CS060, CS010	B	IB
S080	Remove excess structural material	CS011, CS031, CS074, CS083	B	IB
S081	Hollow Structural elements with integrated systems	CS031	B, C	IB
S082	Cobiax technology	CS085	B	IB, WS
S083	Thin-shell structure	CS020, CS061, CS087, CS089	B	IB
S084	Lightweight Structure	CS034, CS072	B	IB
S085	Shell lace structure	CS083	B	IB
S086	Branching columns	CS032	B	IB
S087	Irregular steel trusses structure	CS012	B	IB
S088	Curved diagrid steel envelope structure	CS044	B	IB
S089	Radial bifurcating ribs	CS058, CS063, CS106	B	IB
S090	Multidimensional curvature structure	CS096	B	IB
S091	Skin as Structure	CS074	B	IB
S092	Barrel structure	CS056	B	IB
S093	Responsive adjusting to loads. Infrastructure senses structural compromises and alters structure to compensate	CS028	U	IB, GD
S094	Flexible structures for high wind loads	CS046, CS011	B	IB
S095	Folding Structure	CS033, CS139	C	IB
S096	Suspension structure	CS057	B	IB
S097	Suspended-cable structure	CS059	B	IB
S098	load bearing curvilinear walls	CS084	B	IB
S099	Locally available materials	CS086, CS088	B	IB
S100	Recycled construction materials	CS078	B	IB, WS
S101	Design for less maintenance	CS007, CS015, CS029, CS030, CS055, CS108, CS118, CS119, CS122	C	IB
S102	Photocatalytic cement, neutralize organic and inorganic pollutants. It makes surfaces self-cleaning. Savings in maintenance costs	CS122	B, U	IB
S103	Smart Vapor Retarder blocks	CS136	C	IB
S104	surfaces that inhibit bacterial growth on high-touch surfaces	CS118	C	IB
S105	Non emissive materials	CS135	U	IB, AQ
S106	Self-cleaning paints	CS007	C	IB
S107	Self-cleaning solar panels	CS108	C	IB, EC
S108	Self-cleaning clay roofs	CS119	C	IB
S109	Self-cleaning urban elements	CS055	C	IB
S110	Self-healing cement/concrete	CS015, CS029	C	IB
S111	Industrial Ecology	CS069, CS105	U	WS, EC
S112	Closed-loop models/Cradle-to-cradle	CS026, CS035, CS047, CS069	B, U	GD, WS
S113	Organic Waste to Biogas and fertilizers	CS026, CS070, CS075	U	WS, EC, FD
S114	Biogas to energy (from landfills and waste treatment plants)	CS104	U	WS, EC
S115	Thermal waste treatment plant for (non-recyclables)	CS104	U	WS
S116	Fermentation of Bioorganic waste to energy	CS104	U	WS, EC
S117	Zero waste to landfill	CS047, CS135	U	WS
S118	Design out waste	CS103, CS104	U	WS
S119	Onsite waste recycling	CS036	U	WS
S120	Upcycle/recycle waste	CS078, CS103, CS104	B	WS, GD
S121	Zero Waste	CS135	U	WS, GD
S122	(Net) zero emissions	CS036, CS047, CS064, CS077, CS104, CS135	U	AQ, EC
S123	Non-toxic VOC-free wood glue	CS043	C	AQ, IB
S124	Biofilters for air purification	CS079, CS121	U	AQ
S125	Nature-based solution (NBS) and Biophilia	CS064, CS068	U	BG
S126	Green walls/vertical garden	CS064, CS085	B	BG, IB, EC, AQ
S127	Gravity driven irrigation	CS085	B	BG, WR
S128	Smart irrigation (soil sensors)	CS115	U	WR, GD
S129	Green Roofs	CS064, CS114, CS115	U	BG, IB, EC, AQ
S130	Organic suspended roof gardens	CS036, CS115	U	FD, BG, EC
S131	(Pervious) green corridor/green belt	CS003, CS027, CS64, CS104	U	BG, AQ
S132	Green Infrastructure	CS064	U	BG
S133	Trees and Shrubs	CS064	U	BG, AQ
S134	Permeable (Pervious) Paving/Urban Surfaces	CS064, CS003	U	IB, WR
S135	Recycle/Purify all Urban Water	CS064	U	WR
S136	Bioswales	CS114	U	WR
S137	Protect native landscapes/forests	CS104	U	BG
S138	interconnect protected landscape areas with biotopes	CS104	U	BG
S139	Urban landscapes	CS104	U	BG, IB, AQ
S140	Nature sensitive farming	CS135	U	FD, BG
S141	UV-reflective coating that mitigates bird collisions	CS120	C	IB, BG
S142	Design for increased biodiversity	CS035, CS073	U	BG
S143	Ecosystem Services	CS014, CS067	U	GD
S144	Ecological Performance Standards (EPS)	CS003, CS014, CS067, CS111, CS112	U	GD
S145	Food forest	CS135	U	FD
S146	Fish pond	CS135	U	FD
S147	Edible plants	CS135	U	FD
S148	Water Neutrality	CS135	U	WR
S149	Fog water collection	CS020, CS021, CS024, CS035, CS042, CS107, CS129	B	WR
S150	Rainwater Collection	CS042, CS077, CS114, CS115, CS129, CS 135,	C	WR
S151	Rainwater filtration	CS114	U	WR
S152	Rainwater Storage	CS008, CS114, CS115	B	WR
S153	Water banking (inter-seasonal water storage)	CS115, CS003	U	WR
S154	Cistern Rainwater Storage	CS114, CS115	B	WR
S155	Rainwater storage pockets on façade	CS130	C	WR
S156	Rainwater onsite use	CS114, CS135	U	WR
S157	Greywater onsite use for irrigation and toilet flush	CS076, CS077, CS114, CS135	B	WR
S158	Recharge Aquifers	CS027	U	WR
S159	Connect water infrastructure to the surrounding watershed	CS115	U	WR
S160	water conservation	CS104	U	WR
S161	Adapt rain screens on buildings to enhance evapotranspiration and reduce runoff	CS017	U	WR, IB
S162	multipath low-grade channel designs of underground stormwater infrastructures and street layouts take a similar form	CS003	U	WR
S163	Redirect water to increased flow paths	CS003	U	WR
S164	Eliminate chemical runoff to waterbodies	CS135	U	WR
S165	Membrane filtration technology for safe drinking water	CS041	C	WR
S166	Onsite wastewater treatment (Bioreactor membrane)	CS114	U	WR
S167	Chemical-free wastewater treatment and filtering system	CS019, CS026, CS027, CS038, CS073	U	WR
S168	Wetland	CS135	U	WR, BG
S169	Electric Transport	CS123	U	MT, EC, AQ
S170	Routing Algorithm	CS125	U	MT, EC
S171	Reduced-traffic zones	CS104	U	MT, AQ
S172	Direct Access to public transport	CS104	U	MT
S173	Pedestrian traffic	CS104	U	MT
S174	Connecting public transport to bike lane network	CS104	U	MT
S175	Reduce distance to nearest bus/tram stop	CS104	U	MT
S176	High-density public transport	CS104	U	MT
S177	bicycle networks	CS104	U	MT
S178	Design infrastructure to mimic capacity hierarchies, bifurcation angles, and minimal disruption of flow	CS066	U	MT
S179	Bullet train	CS071	U	MT, EC
S180	Sensors and Actuators	CS028	U	GD
S181	Real-time building energy use auditing	CS104	U	GD, EC
S182	Real-time building CO2 emissions auditing	CS104	U	GD, EC
S183	Integrated systems	CS135	U	GD
S184	self-sustaining off-grid system (energy, water)	CS036, CS081, CS127, CS137	U	GD

**Table 6:** Biomimetic Strategies Matrix Across Scales and City Systems.

	Urban Scale (U)	Whole Building Scale (B)	Building Component Scale (C)
**Energy and Carbon** **(EC)**	S002, S003, S004, S005, S006, S009, S010, S011, S012, S013, S015, S019, S020, S021, S022, S023, S024, S025, S026, S029, S035, S036, S037, S038, S050, S065, S076, S111, S113, S114, S116, S122, S129, S130, S169, S170, S179, S181, S182	S007, S014, S016, S018, S029, S030, S031, S032, S034, S039, S040, S041, S042, S043, S045, S046, S047, S048, S049, S052, S054, S055, S056, S061, S062, S064, S073, S074, S126	S001, S003, S008, S016, S017, S027, S028, S033, S044, S047, S051, S053, S055, S057, S058, S059, S060, S062, S073, S107
**Water** **(WR)**	S128, S134, S135, S136, S148, S151, S153, S156, S158, S159, S160, S161, S162, S163, S164, S166, S167, S168	S127, **S149**, S152, S154, S157	S150, S155, S165
**Waste** **(WS)**	S111, S112, S113, S114, S115, S116, S117, S118, S119, S121	S082, S100, S112, S120	
**Mobility and Transport ** **(MT)**	S065, S169, S170, S171, S172, 173, S174, S175, S176, S177, S178, S179		
**Infrastructure and Buildings ** **(IB)**	S019, S020, S021, S022, S024, S025, S038, S050, S072, S076, S078, S093, S102, S105, S129, S134, S139, S161	S007, S042, S043, S045, S046, S047, S048, S049, S052, S062, S064, S066, S067, S068, S070, S071, S073, S074, S075, S079, S080, S081, S082, S083, S084, S085, S086, S087, S088, S089, S090, S091, S092, S094, S096, S097, S098, S099, S100, S102, S126	S001, S044, S047, S051, S062, S063, S069, S073, S081, S095, S101, S103, S104, S106, S107, S108, S109, S110, S123, S141
**Food** **(FD)**	S113, S130, S140, S145, S146, S147		
**Air Quality** **(AQ)**	S065, S105, S122, S124, S129, S131, S133, S139, S169, S171	S040, S041, S126,	S123
**Governance and Data (GD)**	S077, S093, S112, S121, S128, S143, S144, S180, S181, S182, S183, S184	S016, S049, S054, S061, S112, S120	S016, S017, S053, S059
**Biodiversity and Green Infrastructure (BG)**	S011, S125, S129, S130, S131, S132, S133, S137, S138, S139, S140, S142, S168	S126, S127	S141

## Data Availability

The data presented in this study are available on request from the corresponding author.

## References

[1] UN (2014). World Urbanization Prospects. The 2014 Revision-Highlights.

[2] Chayaamor-Heil N., Hannachi-Belkadi N. (2017). Towards a platform of investigative tools for biomimicry as a new approach for energy-efficient building design. Buildings.

[3] Nishant R., Kennedy M., Corbett J. (2020). Artificial intelligence for sustainability: Challenges, opportunities, and a research agenda. Int. J. Inf. Manag..

[4] Benyus J.M. (1997). Biomimicry: Innovation Inspired by Nature.

[5] Reed B. (2007). Forum: Shifting from ‘sustainability’ to regeneration. Build. Res. Inf..

[6] Pedersen Zari M., Jenkin S. Re-defining cutting edge sustainable design: From eco-efficiency to regenerative development. Proceedings of the Sustainable Building Conference (SB10).

[7] Zari M.P. (2018). Regenerative Urban Design and Ecosystem Biomimicry.

[8] Hunt J. (2004). How can cities mitigate and adapt to climate change?. Build. Res. Inf..

[9] Matyas D., Pelling M. (2015). Positioning resilience for 2015: The role of resistance, incremental adjustment and transformation in disaster risk management policy. Disasters.

[10] Meerow S., Newell J.P., Stults M. (2016). Defining Urban Resilience: A Review.

[11] Folke C. (2006). Resilience: The emergence of a perspective for social-ecological systems analyses. Glob. Environ. Change.

[12] Klein R.J.T., Nicholls R.J., Thomalla F. (2003). Resilience to natural hazards: How useful is this concept?. Environ. Hazards.

[13] Meerow S., Newell J.P. (2015). Resilience and Complexity: A Bibliometric Review and Prospects for Industrial Ecology. J. Ind. Ecol..

[14] Brand F.S., Jax K. (2007). Focusing the Meaning(s) of Resilience: Resilience as a Descriptive Concept and a Boundary Object. Ecol. Soc..

[15] Vale L.J. (2014). The politics of resilient cities: Whose resilience and whose city?. Build. Res. Inf..

[16] Dicken P. (2007). Global Shift: Mapping the Changing Contours of the World Economy.

[17] Armitage D., Johnson D. (2006). Can Resilience be Reconciled with Globalization and the Increasingly Complex Conditions of Resource Degradation in Asian Coastal Regions?. Ecol. Soc..

[18] Elmqvist T., Barnett G., Wilkinson C. (2014). Exploring urban sustainability and resilience. Resilient Sustainable Cities.

[19] Alberti M., Marzluff J.M., Shulenberger E., Bradley G., Ryan C., Zumbrunnen C. (2003). Integrating humans into ecology: Opportunities and challenges for studying urban ecosystems. Bioscience.

[20] Pickett S.T., Cadenasso M.L., McGrath B. (2013). Resilience in Ecology and Urban Design: Linking Theory and Practice for Sustainable Cities. Future City.

[21] (2007). Resilience Alliance, "Urban resilience research prospectus," Canberra, Australia; Phoenix, USA; Stockholm, Sweden. http://www.resalliance.org/files/1172764197_urbanresilienceresearchprospectusv7feb07.pdf.

[22] Keating C., Rogers R., Unal R., Dryer D., Sousa-Poza A., Safford R., Peterson W., Rabadi G. (2003). System of systems engineering. EMJ-Eng. Manag. J..

[23] Hodson M., Marvin S. (2010). Can cities shape socio-technical transitions and how would we know if they were?. Res. Policy.

[24] Seitzinger S.P., Svedin U., Crumley C.L., Steffen W., Abdullah S.A., Alfsen C., Broadgate W.J., Biermann F., Bondre N.R., Dearing J.A. (2012). Planetary stewardship in an urbanizing world: Beyond city limits. AMBIO.

[25] Desouza K.C., Flanery T.H. (2013). Designing, planning, and managing resilient cities: A conceptual framework. Cities.

[26] Pedersen Zari M. Biomimetic Approaches to Architectural Design for Increased Sustainability. Proceedings of the SB07 NZ Sustainable Building Conference.

[27] Bar-Cohen Y. (2005). Biomimetics: Mimicking and inspired-by biology. Smart Structures and Materials 2005: Electroactive Polymer Actuators and Devices (EAPAD).

[28] Webb S. (2005). The Integrated Design Process of CH_2_. Source Environ. Des. Guide.

[29] Zari M.P. (2010). Biomimetic design for climate change adaptation and mitigation. Arch. Sci. Rev..

[30] Biomimicry Institute “Nature’s Unifying Patterns,” Biomimicry Toolbox. https://toolbox.biomimicry.org/core-concepts/natures-unifying-patterns/.

[31] Pacheco-Torgal F., Torgal F.P., Labrincha J.A., Diamanti M.V., Yu C.-P., Lee H.K. (2015). Biotechnologies and Biomimetics for Civil Engineering.

[32] Aldersey-Williams H. (2004). Towards biomimetic architecture. Nat. Mater..

[33] Uchiyama Y., Blanco E., Kohsaka R. (2020). Application of biomimetics to architectural and urban design: A review across scales. Sustainability.

[34] Shimomura M. (2015). New trend of biomimetics: Innovative material technology towards sustainability. Eng. Mater..

[35] Vincent J.F.V., Bogatyreva O.A., Bogatyrev N.R., Bowyer A., Pahl A.K. (2006). Biomimetics: Its practice and theory. R. Soc..

[36] Garcia-Holguera M., Clark O.G., Sprecher A., Gaskin S. (2016). Ecosystem biomimetics for resource use optimization in buildings. Build. Res. Inf..

[37] Verbrugghe N., Rubinacci E., Khan A.Z. (2023). Biomimicry in Architecture: A Review of Definitions, Case Studies, and Design Methods. Biomimetics.

[38] U.N (2017). General Assembly, “Resolution adopted by the General Assembly on 6 July 2017: Work of the Statistical Commission pertaining to the 2030 Agenda for Sustainable Development.

[39] Gong W., Lyu H. Sustainable City Indexing: Towards the Creation of an Assessment Framework for Inclusive and Sustainable Urban-Industrial Development. https://www.unido.org/sites/default/files/files/2018-02/BRIDGE%20for%20Cities_Issue%20Paper_2.pdf.

[40] López M., Rubio R., Martín S., Croxford B. (2017). How Plants Inspire Façades. From Plants to Architecture: Biomimetic Principles for the Development of Adaptive Architectural Envelopes.

[41] Mathews F. (2011). Towards a deeper philosophy of biomimicry. Organ. Environ..

[42] Al-Obaidi K.M., Ismail M.A., Hussein H., Rahman A.M.A. (2017). Biomimetic Building Skins: An Adaptive Approach.

[43] Yuan Y., Yu X., Yang X., Xiao Y., Xiang B., Wang Y. (2017). Bionic Building Energy Efficiency and Bionic Green Architecture: A Review.

[44] Anzaniyan E., Alaghmandan M., Koohsari A.M. (2022). Design, fabrication and computational simulation of a bio-kinetic façade inspired by the mechanism of the Lupinus Succulentus plant for daylight and energy efficiency. Sci. Technol. Built Environ..

[45] Blau M.L., Luz F., Panagopoulos T. (2018). Urban river recovery inspired by nature-based solutions and biophilic design in Albufeira, Portugal. Land.

[46] Hayes S., Desha C., Burke M., Gibbs M., Chester M. (2019). Leveraging socio-ecological resilience theory to build climate resilience in transport infrastructure. Transp. Rev..

[47] Ahamed M.K., Wang H., Hazell P.J. (2022). From Biology to Biomimicry: Using Nature to Build Better Structures—A Review.

[48] Buck N.T. (2017). The art of imitating life: The potential contribution of biomimicry in shaping the future of our cities. Environ. Plan. B Urban Anal. City Sci..

[49] Radwan G.A.N., Osama N. (2016). Biomimicry, an Approach, for Energy Effecient Building Skin Design. Procedia Environ. Sci..

[50] Hayes S., Desha C., Baumeister D. (2020). Learning from nature—Biomimicry innovation to support infrastructure sustainability and resilience. Technol. Forecast. Soc. Change.

[51] Zari M.P., Hecht K. (2020). Biomimicry for regenerative built environments: Mapping design strategies for producing ecosystem services. Biomimetics.

[52] Gruber P., Imhof B. (2017). Patterns of growth-biomimetics and architectural design. Buildings.

[53] Badarnah L. (2015). A Biophysical Framework of Heat Regulation Strategies for the Design of Biomimetic Building Envelopes. Procedia Engineering.

[54] Chou J.S., Ngo N.T., Chong W.K., Gibson G.E. (2016). Big data analytics and cloud computing for sustainable building energy efficiency. Start-Up Creation: The Smart Eco-Efficient Built Environment.

[55] Zari M.P., Storey J. (2007). An ecosystem based biomimetic theory for a regenerative built environment. Lisbon Sustainable Building Conference (SB07).

[56] Montana-Hoyos C., Fiorentino C. (2016). Bio-Utilization, Bio-Inspiration and Bio-Affiliation in Design for Sustainability. Int. J. Des. Objects.

[57] Blanco E., Zari M.P., Raskin K., Clergeau P. (2021). Urban ecosystem-level biomimicry and regenerative design: Linking ecosystem functioning and urban built environments. Sustainability.

[58] Ilieva L., Ursano I., Traista L., Hoffmann B., Dahy H. (2022). Biomimicry as a Sustainable Design Methodology—Introducing the ‘Biomimicry for Sustainability’ Framework. Biomimetics.

[59] Badarnah L. (2016). Light Management Lessons from Nature for Building Applications. Procedia Engineering.

[60] Dash S.P. (2018). Application of biomimicry in building design. Int. J. Civ. Eng. Technol..

[61] Jamei E., Vrcelj Z. (2021). Biomimicry and the built environment, learning from nature’s solutions. Appl. Sci..

[62] Kadar T., Kadar M. Sustainability Is Not Enough: Towards AI Supported Regenerative Design. Proceedings of the 2020 IEEE International Conference on Engineering, Technology and Innovation (ICE/ITMC).

[63] Spiegelhalter T., Arch R.A. (2010). Biomimicry and circular metabolism for the cities of the future. WIT Trans. Ecol. Environ..

[64] Lazarus M.A., Crawford C. (2011). Returning genius to the place. Archit. Des..

[65] Sommese F., Badarnah L., Ausiello G. (2022). A Critical Review of Biomimetic Building Envelopes: Towards a Bio-Adaptive Model from Nature to Architecture.

[66] Pedersen Zari M. (2009). An architectural love of the living: Bio-inspired design in the pursuit of ecological regeneration and psychological well-being. WIT Trans. Ecol. Environ..

[67] Dicks H., Bertrand-Krajewski J.L., Ménézo C., Rahbé Y., Pierron J.P., Harpet C. (2021). Applying Biomimicry to Cities: The Forest as Model for Urban Planning and Design. Philosophy of Engineering and Technology.

[68] Faragalla A.M.A., Asadi S. (2022). Biomimetic Design for Adaptive Building Façades: A Paradigm Shift towards Environmentally Conscious Architecture. Energies.

[69] Imani N., Vale B. (2022). Developing a Method to Connect Thermal Physiology in Animals and Plants to the Design of Energy Efficient Buildings. Biomimetics.

[70] Faragllah R.N. (2021). Biomimetic approaches for adaptive building envelopes: Applications and design considerations. Civ. Eng. Archit..

[71] Benyus J., Dwyer J., El-Sayed S., Hayes S., Baumeister D., Penick C.A. (2022). Ecological performance standards for regenerative urban design. Sustain. Sci..

[72] Elshapasy R.A.I., Ibrahim M.A., Elsayad Z. (2022). Bio-tech Retrofitting to Create a Smart-Green University. Sustain. Dev. Plan. XII.

[73] Hao X., Novotny V., Nelson V. (2010). Water Infrastructure for Sustainable Communities: China and the World.

[74] Movva S.H., Velpula S.L. (2020). An analytical approach to sustainable building adaption using biomimicry. Materials Today: Proceedings.

[75] Oguntona O.A., Aigbavboa C.O. (2019). Assessing the awareness level of biomimetic materials and technologies in the construction industry. IOP Conference Series: Materials Science and Engineering.

[76] Quintero A., Zarzavilla M., Tejedor-Flores N., Mora D., Austin M.C. (2021). Sustainability assessment of the anthropogenic system in panama city: Application of biomimetic strategies towards regenerative cities. Biomimetics.

[77] Speck O., Möller M., Grießhammer R., Speck T. (2022). Biological Concepts as a Source of Inspiration for Efficiency, Consistency, and Sufficiency. Sustainability.

[78] Widera B. Biomimetic and Bioclimatic Approach to Contemporary Architectural design on the Example of CSET Building. Proceedings of the International Multidisciplinary Scientific GeoConference: SGEM.

[79] AlAli M., Mattar Y., Alzaim M.A., Beheiry S. (2023). Applications of Biomimicry in Architecture, Construction and Civil Engineering. Biomimetics.

[80] Aslan D., Selçuk S.A., Avinç G.M. (2022). A Biomimetic Approach to Water Harvesting Strategies: An Architectural Point of View. Int. J. Built Environ. Sustain..

[81] Del Rosario M.D.L.Á.O., Beermann K., Austin M.C. (2023). Environmentally Responsive Materials for Building Envelopes: A Review on Manufacturing and Biomimicry-Based Approaches. Biomimetics.

[82] Elsakksa A., Marouf O., Madkour M. (2022). Biomimetic Approach for Thermal Performance Optimization in Sustainable Architecture. Case study: Office Buildings in Hot Climate Countries. IOP Conference Series: Earth and Environmental Science.

[83] Mazzoleni I., Barthakur A., Price S., Zajfen V., Varma S., Mehlomakulu B., Portillo H., Milner S. (2008). Eco-systematic restoration: A model community at Salton Sea. WIT Trans. Ecol. Environ..

[84] Sharma V., Singh P.K. (2021). Protecting humanity by providing sustainable solution for mimicking the nature in construction field. Materials Today: Proceedings.

[85] Van den Dobbelsteen A.A.J.F., Keeffe G., Tillie N.M.J.D., Roggema R.E. (2021). Cities as organisms: Using biomimetic principles to become energetically self-supporting and climate-proof. Proceedings of the First International Conference on Sustainable Urbanization (ICSU 2010).

